# Oral glucose absorption is enhanced in early metabolic dysfunction-associated steatotic liver disease

**DOI:** 10.1007/s00125-026-06730-5

**Published:** 2026-04-21

**Authors:** Domenico Tricò, Tongzhi Wu, Noemi Cimbalo, Martina Chiriacò, Cong Xie, Luca Sacchetta, Lorenzo Nesti, Simone Gallo, Lorenza Santoni, Maria C. Masoni, Giovanni Petralli, Teresa V. Fiorentino, Roberto Bizzotto, Maria T. Scozzaro, Silvia Frascerra, Simona Baldi, Maurizia R. Brunetto, Andrea Mari, Christopher K. Rayner, Andrea Natali

**Affiliations:** 1 Department of Clinical and Experimental Medicine, University of Pisa, Pisa, Italy; 2 Laboratory of Metabolism, Nutrition and Atherosclerosis, University of Pisa, Pisa, Italy; 3 School of Medicine and Centre of Research Excellence in Translating Nutritional Science to Good Health, College of Health, Adelaide University, Adelaide, SA Australia; 4 Department of Surgical, Medical and Molecular Pathology and Critical Care Medicine, University of Pisa, Pisa, Italy; 5 Institute of Life Sciences, Sant’Anna School of Advanced Studies, Pisa, Italy; 6 Department of Clinical and Molecular Medicine, University of Rome La Sapienza, Rome, Italy; 7 Institute of Neuroscience, National Research Council, Padua, Italy

**Keywords:** Fatty liver, Gastric emptying, Glucose intolerance, Insulin resistance, Intestinal glucose absorption, Obesity, Sodium–glucose cotransporter, The metabolic syndrome

## Abstract

**Aims/hypothesis:**

Hepatic glucose flux plays a crucial role in the progression of metabolic dysfunction-associated steatotic liver disease (MASLD), promoting de novo lipogenesis, inflammation and fibrosis. This study aimed to evaluate the kinetics of oral glucose absorption and one of its key modulators, gastric emptying, in individuals with early-stage MASLD vs matched control individuals.

**Methods:**

We quantified glucose metabolic fluxes during a 75 g OGTT using stable isotopes in individuals with MASLD without fibrosis and in healthy control individuals. In a separate cohort, we measured the gastric emptying rate using the ^13^C-acetate breath test during an OGTT and estimated hepatic steatosis risk.

**Results:**

Compared with the control group, in the MASLD group the rate of appearance of oral ingested glucose (RaO) normalised to body weight was 34% higher at 1 h post-OGTT (+318±142 µmol/kg, *p*=0.031), resulting in a 52% increase in total glucose absorption (+6.4±1.8 g, *p*=0.001). Participants with MASLD exhibited reduced glucose clearance relative to plasma insulin levels but preserved post-load suppression of endogenous glucose production, indicating peripheral rather than hepatic insulin resistance. Among glucose metabolic fluxes, RaO showed the strongest association with prevalent MASLD, with each 1-SD increase in 1 h RaO being associated with fivefold higher odds of MASLD (OR 4.99 [95% CI 1.44, 31.57], *p*=0.036), independent of potential confounders. Gastric emptying rate was not associated with hepatic steatosis risk.

**Conclusions/interpretation:**

Oral glucose absorption is augmented in individuals with MASLD without fibrosis, apparently unrelated to accelerated gastric emptying. This metabolic alteration may represent an early driver of MASLD pathogenesis, preceding hepatic insulin resistance. Future research should investigate whether modulation of intestinal glucose absorption confers therapeutic benefits in MASLD.

**Graphical Abstract:**

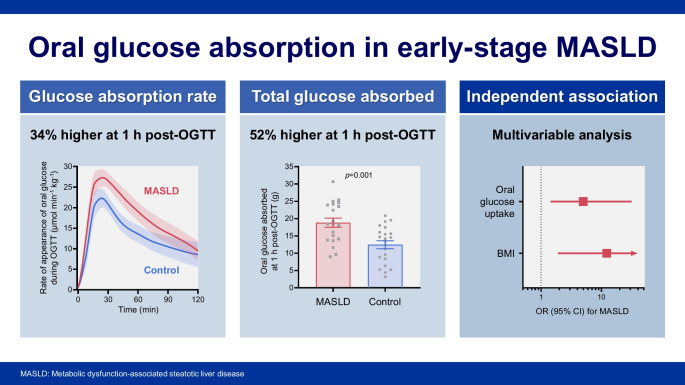

**Supplementary Information:**

The online version contains peer-reviewed but unedited supplementary material available at 10.1007/s00125-026-06730-5.


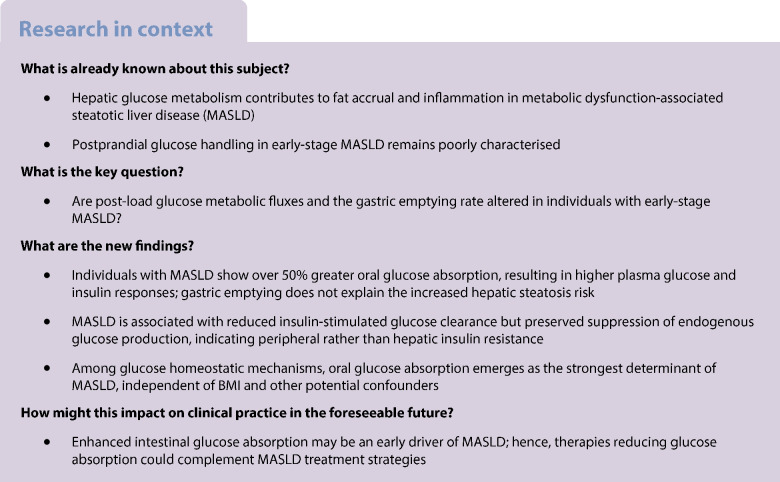


## Introduction

Metabolic dysfunction-associated steatotic liver disease (MASLD) poses a growing health challenge, affecting approximately 40% of adults globally and conferring substantially increased risks of hepatocellular carcinoma and cardiovascular mortality [1]. Formerly termed non-alcoholic fatty liver disease (NAFLD), MASLD exists on a spectrum ranging from simple steatosis to steatohepatitis, fibrosis and cirrhosis [2]. The pathogenesis of MASLD is multifactorial and intrinsically linked to metabolic derangements, with chronic hyperglycaemia, insulin resistance and beta cell dysfunction recognised as key drivers of hepatic lipid accumulation and inflammation [3, 4].

Emerging evidence from both clinical and experimental research has hinted at profound alterations in oral glucose handling in individuals with MASLD, supporting a role for enhanced postprandial glucose flux to the liver in promoting de novo lipogenesis (DNL), inflammation and fibrosis. Oral glucose absorption, quantified in vivo by the rate of appearance of oral ingested glucose (RaO), is a major determinant of postprandial glycaemic and insulinaemic responses, especially in individuals without diabetes [5, 6]. RaO is primarily regulated by gastric emptying, which determines the rate of entry of ingested glucose into the small intestine [7], and by the expression of glucose carriers in the upper small intestine, particularly sodium–glucose cotransporter 1 (SGLT1) [8], whose expression and activity have been associated with MASLD risk [9–11]. On the other hand, gastrointestinal motility and SGLT1 regulate the glucose-induced release of the incretin hormones glucagon-like peptide-1 (GLP-1) and glucose-dependent insulinotropic polypeptide (GIP) [8], which may mitigate hepatic fat accumulation through improved glucose and lipid homeostasis [12, 13]. The role of postprandial hepatic glucose flux in the pathogenesis of MASLD is further corroborated by the observation that 1 h plasma glucose during an OGTT, influenced by both gastric emptying [14, 15] and intestinal glucose absorption [14, 16], is elevated in people with MASLD and correlates directly with hepatic steatosis and fibrosis severity [17, 18].

Nonetheless, previous studies have enrolled predominantly individuals with advanced MASLD and relied on proxies of oral glucose absorption (e.g. expression of glucose transporters or blood glucose profiles during OGTTs). Thus, a substantial knowledge gap persists regarding in vivo dynamics and determinants of glucose flux, particularly in the ‘early’ stage of MASLD. These limitations impede the development of targeted therapeutic strategies, as oral glucose handling (if confirmed as a key pathogenetic factor) could constitute a modifiable contributor to the progression of MASLD.

This study employed the gold-standard dual-tracer technique, involving both i.v. and oral administration of stable glucose isotopes, to quantify intestinal glucose absorption kinetics after an oral glucose load, while simultaneously assessing peripheral glucose disposal and hepatic glucose production. We further explored, in a separate cohort, the relationship between gastric emptying of an oral glucose load assessed by the ^13^C-acetate breath test and the hepatic steatosis index (HSI), a surrogate marker of liver fat. By delineating post-load glucose fluxes and their underlying mechanisms, we tested the hypothesis that oral glucose absorption is higher in individuals with early-stage MASLD than in matched control individuals.

## Methods

### Study 1: dual-tracer study

#### Participants

Male and female volunteers aged 18–65 years, with a BMI of 18–40 kg/m^2^, representative of the local population, were recruited at the Section of Dietetics and Clinical Nutrition at the University Hospital of Pisa (Italy) during screening for nutrition and pharmacological trials. Recruitment procedures and study protocols were approved by the local Human Ethics Committee (North-West Wide-Area Ethics Committee, protocol no. 13053_NATALI, 6792_NATALI, 8512015_NATALI). At screening, medical history and medication use were recorded using standardised questionnaires, which also captured current and prior alcohol consumption. Volunteers were excluded if they had acute or chronic disorders, including type 2 diabetes, were taking medications influencing glucose or lipid metabolism, or were pregnant or lactating. The study was conducted in accordance with the principles expressed in the Declaration of Helsinki. All participants provided written informed consent before enrolment.

#### MASLD assessment

In accordance with a 2023 multisociety consensus statement [2], steatotic liver disease (SLD) was diagnosed using ultrasound imaging by a trained radiologist, while MASLD was defined as the presence of SLD associated with at least one cardiometabolic risk factor, in the absence of significant alcohol consumption (>140 g/week or >20 g/day for female participants; >210 g/week or >30 g/day for male participants) and other discernible causes of liver disease. Fasting blood samples were collected for measurement of complete blood count, HbA_1c_, total and direct bilirubin, and liver enzymes including aspartate aminotransferase (AST), alanine aminotransferase (ALT), γ-glutamyl transferase and alkaline phosphatase. The ratio between the concentrations of AST and ALT (AST/ALT ratio) was calculated. Individuals with no evidence of liver fibrosis of any grade, as assessed using the Fibrosis-4 (FIB-4) index [19], were included in the study. The FIB-4 index was calculated as age (years) × AST (U/l) / (platelet [count in 10^9^/l] × √(ALT [U/l]), and liver fibrosis was excluded based on a cutoff of 1.3 [19]. In six cases with a FIB-4 score ≥1.3 (up to 1.8), fasting liver elastography was performed by a trained hepatologist using a FibroScan device (EchoSens, Paris, France), and the absence of significant fibrosis was confirmed by liver stiffness values ≤8 kPa, as indicated by the current clinical guidelines for MASLD management [20].

#### Metabolic tests

**Fig. 1:**
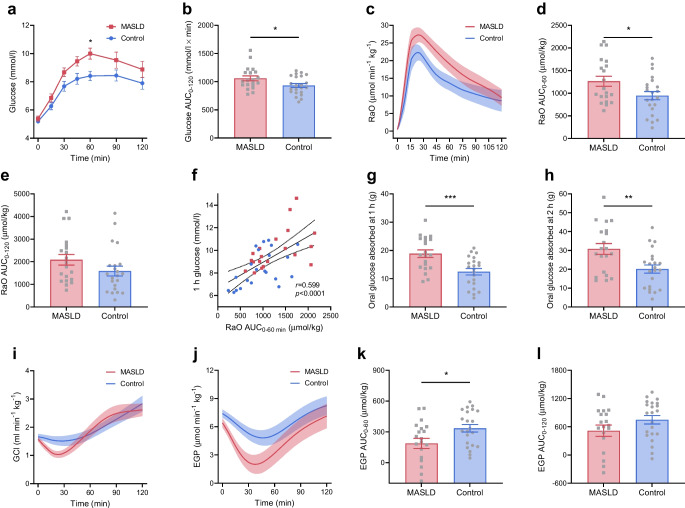
Plasma glucose and glucose metabolic fluxes. (**a**–**e**, **g**–**l**) Profiles and AUCs during a 75 g OGTT of plasma glucose (**a**, **b**), tracer-derived RaO (**c**–**e**), total oral glucose absorbed at 1 h and 2 h (**g**, **h**), GCl (**i**) and EGP (**j**–**l**) in individuals with early-stage MASLD (*n*=20) and control individuals (*n*=22). Data are mean ± SEM indicated by error bars or shaded areas. (**f**) Correlation of RaO with 1 h plasma glucose, evaluated using Pearson correlation. Best fit line (continuous line) and 95% CIs (dotted lines) are shown. Group differences were analysed using Student’s *t* test. Repeated measures were analysed by two-way ANOVA followed by multiple pairwise comparisons using Sidak’s post hoc tests. *p<0.05, **p<0.01, ***p<0.001

Each participant underwent a frequently sampled, 75 g OGTT, modified with the use of two stable glucose isotopes to quantify glucose metabolic fluxes. At 08:00 hours, after a standardised dinner and an overnight fast (12 h), participants were admitted to our Clinical Research Unit. A 20-gauge polyethylene cannula was inserted into an antecubital vein for the infusion of all test substances. A second cannula was inserted retrogradely into a wrist vein for blood sampling, and the hand was kept wrapped in a heated blanket to achieve the arterialisation of venous blood. From time −120 min, volunteers received a 5 h primed (28 µmol/kg) continuous (0.28 µmol min^−1^ kg^−1^) infusion of 6,6-[^2^H_2_]glucose (Cambridge Isotope Laboratories, Tewksbury, MA, USA). After 2 h (time 0 min), participants consumed a 300 ml oral glucose drink containing 73.5 g dextrose and 1.5 g [U-^13^C]glucose (Cambridge Isotope Laboratories) within 5 min (time 0–5 min). Arterialised blood samples were collected throughout the test at times −120, −15, 0, 15, 30, 45, 60, 90 and 120 min to measure plasma glucose, insulin, C-peptide, glucagon, GLP-1, GIP, NEFA and glucose tracer enrichment (electronic supplementary material [ESM] Fig. 1). Participants were classified as having impaired fasting glucose (IFG) or impaired glucose tolerance (IGT) according to current diagnostic criteria [21].

#### Analytical procedures

Routine biochemical analyses were performed at the core laboratory of the University Hospital of Pisa, while other analyses were performed at the Laboratory of Nutrition, Metabolism, and Atherosclerosis of the University of Pisa. Plasma glucose concentrations during the OGTT were measured immediately by the glucose-oxidase technique on a GM9 Glucose Analyser (Analox Instruments, Stourbridge, UK). 6,6-[^2^H_2_]glucose and [U-^13^C]glucose were measured by GC-MS [22]. Insulin and C-peptide were assayed by electrochemiluminescence on a COBAS e411 instrument (Roche, Indianapolis, IN, USA). Glucagon was measured by ELISA (Mercodia, Uppsala, Sweden). Total GLP-1 and GIP were measured by ELISA (Merck, Darmstadt, Germany). NEFA were assayed by standard spectrophotometric methods on a Synchron UniCel DxC 600 instrument (Beckman Instruments).

#### Glucose kinetics

RaO, whole-body glucose clearance (GCl) and endogenous glucose production (EGP) were assessed from the time course of the plasma tracer/tracee ratio of 6,6-[^2^H_2_]glucose and [U-^13^C]glucose using a previously described model [23]. Data were normalised by individual body weight to facilitate comparison with prior studies and to account for group differences in BMI.

#### Beta cell function modelling

Insulin secretion rate (ISR) was estimated by C-peptide deconvolution [24]. Beta cell function parameters were calculated by mathematical modelling of ISR and glucose concentrations, as previously reported [25, 26]. The relationship between glucose and ISR is described as the sum of two components, with the first component representing the dependence of ISR on absolute glucose concentration. The quasi-linear dose–response function relating the two variables is described by a slope, named beta cell glucose sensitivity (β-GS), and by ISR at a fixed glucose concentration of 5 mmol/l (ISR@5). This function can be modulated by several factors (i.e. gastrointestinal hormones, neurotransmitters, non-glucose substrates), which are collectively modelled as a potentiation factor. The potentiation factor is set to be a positive function of time and to average the value 1 during the OGTT. The potentiation factor ratio of the values at 100–120 min vs 0–20 min is used to express this component. The second component of insulin secretion represents the dynamic dependence of ISR on the rate of change of glucose concentration and is named beta cell rate sensitivity (β-RS).

#### Insulin clearance

Fasting and post-load endogenous insulin clearance, which refers to the process (mainly hepatic) by which the secreted insulin is removed from the bloodstream [27], were calculated as the ratios between fasting levels or AUC, respectively, of ISR and plasma insulin, allowing comparisons with previous studies [5, 28].

#### Insulin sensitivity

Whole-body and hepatic insulin sensitivity were quantified using glucose kinetics data, as previously reported [16]. Whole-body insulin sensitivity was measured as the GCl normalised to plasma insulin levels, calculated as the ratio between fasting GCl and plasma insulin (fasting GCl/Ins) or between their AUC during the OGTT (GCl/Ins AUC_0–120_). Hepatic insulin resistance was measured as EGP × ISR, the latter being a proxy of portal insulin levels, calculated as fasting EGP multiplied by fasting ISR (fasting EGP × ISR) or by the product of their AUC during the OGTT (EGP × ISR AUC_0–120_). Additionally, to facilitate comparison with prior studies, we calculated surrogate indices of whole-body, hepatic and adipose tissue insulin sensitivity. Whole-body insulin sensitivity was estimated using both the HOMA-IR and the Matsuda index [29]. Hepatic insulin resistance was estimated using the hepatic insulin resistance index (HIRI) (calculated as glucose AUC_0–30_ [mmol/l × h] × insulin AUC_0–30_ [pmol/l × h] [29]). Adipose tissue insulin resistance was estimated using the Adipo-IR index (calculated as fasting NEFA [mmol/l] × fasting insulin [pmol/l] [30]).

### Study 2: gastric emptying and hepatic steatosis

#### Participants

Male and female participants aged 18–70 years, with a BMI of 18–30 kg/m^2^ and no prior history of diabetes mellitus, representative of the local population, were recruited from the community in Adelaide, Australia, via public advertisements. Participants were screened using a 75 g OGTT with concurrent measurement of gastric emptying by stable isotope breath test for inclusion in a low-energy sweetener trial approved by the Central Adelaide Local Health Network Human Research Ethics Committee (protocol n. 2022/HRE00302). Participants were excluded if they reported habitual use of more than one serve per day of any food or beverage containing a low-energy sweetener during the past 3 months, significant gastrointestinal symptoms, a history of gastrointestinal surgery, or a requirement for medication known to affect gastrointestinal function or appetite. Female participants who used oral contraceptives or were pregnant were also excluded. The study was conducted in accordance with the principles expressed in the Declaration of Helsinki. All participants provided written informed consent.

#### Measurement of gastric emptying and blood glucose response to a 75 g OGTT

Participants refrained from strenuous physical activity for 24 h before the study and fasted from food and nutrient beverages from 20:00 hours but were allowed to drink water until midnight, before attending our clinical research facility at the University of Adelaide the following day at 08:00 hours. On arrival, an i.v. cannula was inserted into a forearm vein and the arm kept heated to allow sampling of arterialised venous blood. A fasting sample was collected for measurements of HbA_1c_, blood glucose and biochemical variables, including serum AST, ALT, alkaline phosphatase and γ-glutamyl transferase. Participants then consumed a 300 ml glucose drink containing 75 g glucose and 150 mg ^13^C-acetate within 5 min (time 0–5 min). Additional venous blood was sampled at 30, 60, 120 and 180 min after the drink. Blood glucose concentrations were assessed using a glucometer (Optium Xceed; Abbott Laboratories, USA) and reported as the mean of duplicate measurements at each time point.

Breath samples were collected immediately before the drink, then every 5 min after the drink for the first 30 min and every 15 min for a further 150 min. ^13^CO_2_ in each breath sample was measured by a non-dispersive infrared spectrometer (FANci2; Fischer Analysen Instrumente, Germany). The gastric half-emptying time (T50) and gastric retention at 1 h after the drink were calculated using the Wagner–Nelson method, as previously described [31, 32]. This method has shown comparable accuracy to scintigraphy for the measurement of gastric emptying of both solid and liquid meals [31].

The HSI, a surrogate marker of liver fat, was calculated as follows: 8 × ALT (U/l) /AST (U/l) ratio + BMI (kg/m^2^) + 2 (if female) + 2 (if diabetes) [33]. An HSI score ≥36 is considered high-risk for MASLD, while MASLD is unlikely with HSI <30 [33].

### Statistical analysis

Continuous variables were tested for normality using the Shapiro–Wilk test. Data are reported as mean ± SD or median (IQR) if not normally distributed, unless otherwise stated. AUC was calculated using the trapezoidal rule.

In Study 1, group differences were analysed using unpaired Student *t* test, Mann–Witney test or Fisher exact test, as appropriate. Repeated measures were analysed by two-way ANOVA followed by multiple pairwise comparisons using Sidak’s tests. Relationships were tested by Pearson correlation. Univariable and multivariable logistic regression analysis was used to identify the postprandial glucose homeostatic mechanisms associated with MASLD, selected between early glucose absorption rate (RaO AUC_0–60_), whole-body insulin sensitivity (GCl/Ins AUC_0–120_), hepatic insulin resistance (EGP × ISR AUC_0–120_), beta cell function (β-GS) and insulin clearance. ORs with 95% CIs per SD change in the predictor variable were obtained from estimating models on standardised variables. To account for the potential impact of ageing, sex, glucose management and obesity on glucose metabolic fluxes or MASLD, multivariable models were performed including age, sex (self-reported) and HbA_1c_ as covariates; BMI was introduced only later in subsequent models to minimise the risk of overadjustment, given that glucose fluxes were already normalised to body weight. To disentangle whether observed group differences reflected hepatic steatosis per se or overall adiposity, we also performed subset analyses excluding participants with extreme BMI, either >33 kg/m^2^ (*n*=3, all in the MASLD group) or <20 kg/m^2^ (*n*=3, all in the control group). The resulting cohort subset and analyses were referred to as BMI-matched.

In Study 2, participants were stratified into three subgroups based on the HSI cut-offs of 30 and 36 [33]. Demographic and clinical variables between subgroups with HSI ≥36 and HSI<30 were compared using Fisher’s exact test, unpaired Student *t* tests or Mann–Whitney *U* tests, as appropriate. The T50 and gastric emptying at 1 h post-OGTT were compared in subgroups with HSI ≥36 and HSI <30 using unpaired Student *t* test, both before and after adjustment for age, sex, HbA_1c_ and BMI. The blood glucose response to oral glucose in subgroups with HSI ≥36 and HSI <30 was also compared using two-factor repeated measures ANOVA, with group and time as factors. The relationships between HSI and 1 h blood glucose, T50 or gastric emptying at 1 h post-OGTT were evaluated using Pearson correlation analysis.

Statistical analyses were performed using either JMP SE software version 18.2.1 (JMP Statistical Discovery, Cary, NC, USA) or SPSS statistics version 29.0 (IBM, NY, USA). All tests were conducted at a two-sided α level of 0.05.

## Results

### Study 1

#### Participants

Forty-two volunteers completed the dual-tracer OGTT (mean age 45±14 years, 25 men and 17 women, BMI 26.7±4.6 kg/m^2^, liver enzymes <1.5 × upper limit of normal in all participants). Participants’ clinical and metabolic characteristics are reported in Table 1. Individuals with MASLD (*n*=20, 47.6%) had a similar age, sex distribution and BP to individuals without MASLD. The MASLD group had higher BMI (+3.8±1.3 kg/m^2^, *p*=0.007), HbA_1c_ (+3.2±1.5 mmol/mol [0.3±0.1%], *p*=0.036), LDL-cholesterol (+0.5±0.3 mmol/l, *p*=0.010), triglycerides (+0.8±0.4 mmol/l, *p*=0.0001), ALT (+8±3 U/l, *p*=0.021) and γ-glutamyl transferase (+19±8 U/l, *p*=0.005) levels.

**Table 1:** Clinical and metabolic characteristics of Study 1 participants

Characteristic	MASLD (*n*=20)	Control (*n*=22)	*p* value
Women, *n* (%)	7 (35)	10 (46)	0.543
Age, years	50 (37–58)	44 (28–56)	0.252
BMI, kg/m^2^	28.7±4.4	24.9±4.2	0.007*
Waist circumference, cm	105±8	98±12	0.147
Systolic BP, mmHg	131±8	132±7	0.780
Diastolic BP, mmHg	84±8	80±9	0.433
HbA_1c_, mmol/mol	39±5	35±4	0.036*
HbA_1c_, %	5.7±0.5	5.4±0.4	0.036*
Fasting glucose, mmol/l	5.4±0.8	5.2±0.5	0.353
1 h glucose, mmol/l	10.0±1.8	8.4±1.6	0.004**
2 h glucose, mmol/l	8.9±2.5	7.9±2.0	0.178
Glucose peak, mmol/l	10.5±1.9	9.2±1.9	0.048*
Fasting insulin, pmol/l	85 (55–117)	48 (28–69)	0.0004***
IFG/IGT, *n* (%)	17 (85.0)	14 (63.6)	0.166
Total cholesterol, mmol/l	4.97 (4.48–5.91)	4.40 (3.73–5.10)	0.025*
HDL-cholesterol, mmol/l	1.22 (0.91–1.48)	1.42 (1.04–1.79)	0.144
LDL-cholesterol, mmol/l	3.37 (2.75–4.01)	2.54 (2.02–3.16)	0.010*
Triglycerides, mmol/l	1.38 (0.36–1.85)	0.79 (0.60–0.97)	0.0001***
Creatinine, µmol/l	76.9±10.6	72.5±15.0	0.272
eGFR (CKD-EPI), ml/min per 1.73 m^2^	93±16	105±23	0.056
AST, U/l	20 (18–26)	17 (15–23)	0.058
ALT, U/l	26 (16–32)	16 (14–22)	0.021*
AST/ALT ratio	0.95±0.31	1.18±0.38	0.057
γ-Glutamyl transferase, U/l	30 (17–64)	14 (10–22)	0.005**
Alkaline phosphatase, U/l	64 (46–73)	67 (47–88)	0.233
Total bilirubin, µmol/l	8.9 (6.2–12.7)	7.0 (6.0–11.6)	0.458
Direct bilirubin, µmol/l	3.3 (2.2–5.0)	2.6 (2.2–5.0)	0.649
Platelets, *n*/µl	217 (185–251)	255 (223–283)	0.069
HSI	38.7±5.7	32.8±5.3	0.001**
FIB-4	0.9 (0.6–1.4)	0.7 (0.6–0.9)	0.070

Data are presented as *n* (%), mean ± SD or median (IQR)

^*^p<0.05, **p<0.01, ***p<0.001 (analysed by Fisher’s exact test, Student’s *t* test or Mann–Whitney test, as appropriate)

IFG, impaired fasting glucose; IGT, impaired glucose tolerance

#### Glucose metabolic fluxes

Plasma glucose concentrations at fasting and 2 h post-OGTT were similar between the two groups (Table 1, Fig. 1a, b). However, blood glucose at 1 h post-OGTT (+1.5±0.5 mmol/l, *p*=0.004), peak glucose (+1.2±0.6 mmol/l, *p*=0.048) and glucose AUC_0–120_ (+127±56 mmol/l × min, *p*=0.028) were higher in the MASLD group than in the control group.

The RaO was substantially higher in the MASLD group, particularly in the first hour of the OGTT (Fig. 1c–e), with RaO AUC_0–60_ being 33.6% greater in the MASLD group than in the control group (+318±142 µmol/kg, *p*=0.031) and directly correlating with 1 h plasma glucose (*r*=0.599, *p*<0.0001) (Fig. 1f). Group differences in RaO AUC_0–60_ and RaO AUC_0–120_ were statistically significant after adjustment for age, sex and HbA_1c_ (*p*=0.011 and *p*=0.048, respectively) and after further adjustment for BMI (*p*=0.003 and *p*=0.015, respectively). In the MASLD group compared with the control group, the total amount of absorbed oral glucose was 51.6% higher at 1 h during the OGTT (+6.4±1.8 g, *p*=0.001) and 53.2% higher at 2 h (+10.7±3.6 g, *p*=0.005) (Fig. 1g, h). Results were materially unchanged in BMI-matched sensitivity analyses (ESM Table 1). After excluding participants with extreme BMI values, group differences in BMI disappeared (*p*=0.177), while differences remained statistically significant for RaO AUC_0–60_ (+446±152 µmol/kg, *p*=0.006), RaO AUC_0–120_ (+737±317 µmol/kg, *p*=0.028) and absorbed oral glucose at 1 h (+6.9±2.0 g, *p*=0.001) and 2 h (+11.5±3.8 g, *p*=0.006) during the OGTT. These differences persisted after adjustment for age, sex and HbA_1c_, as well as after further adjustment for BMI (*p*<0.02 for all).

Whole-body plasma GCl was similar between groups during both fasting and the OGTT (Fig. 1i), without significant differences in GCl AUC_0–60_ or GCl AUC_0–120_ in either univariable or multivariable analyses (*p*>0.10).

The EGP was similar between groups during fasting (*p*=0.135) and partially suppressed after the oral glucose load (Fig. 1j–l). In the MASLD group, time course profiles displayed lower EGP during the first hour of the OGTT, with EGP AUC_0–60_ being 44.2% more suppressed (−148.0±62.4 µmol/kg, *p*=0.021) compared with the control group. Group differences in EGP AUC_0–60_ remained statistically significant after adjustment for age, sex and HbA_1c_ (*p*=0.016) and after further adjustment for BMI (*p*=0.014).

#### Beta cell function and insulin clearance

**Fig. 2:**
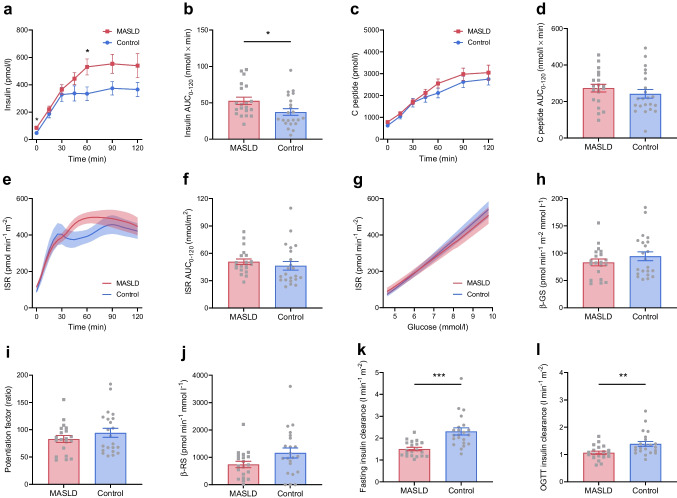
Beta cell function and insulin clearance. (**a**–**f**) Profiles and AUCs during a 75 g OGTT of plasma insulin (**a**, **b**), C-peptide (**c**, **d**) and C-peptide-derived ISR (**e**, **f**) in individuals with early-stage MASLD (*n*=20) and control individuals (*n*=22). (**g**) Dose–response function between the observed plasma glucose concentrations and ISR during the OGTT. (**h**–**j**) Model-derived beta cell function parameters, including β-GS (**h**), potentiation factor (**i**) and β-RS (**j**). (**k**, **l**) Insulin clearance at fasting (**k**) and during the OGTT (**l**) in individuals with early-stage MASLD or control individuals. Data are mean ± SEM indicated by error bars or shaded areas. Group differences were analysed using Student’s *t* test. Repeated measures were analysed by two-way ANOVA followed by multiple pairwise comparisons using Sidak’s post hoc tests. *p<0.05, **p<0.01, ***p<0.001

Plasma insulin levels were higher in the MASLD group than the control group during fasting (+38±10 pmol/l, *p*=0.0004) and throughout the OGTT (AUC_0–120_ +15±7 nmol/l × min, *p*=0.031) (Table 1, Fig. 2a, b). Moreover, individuals with MASLD displayed a higher ISR at fasting (+26±12 pmol min^−1^ m^−2^, *p*=0.039) and at 1 h post-OGTT (+102±48 pmol min^−1^ m^−2^, *p*=0.038), although C-peptide levels (AUC_0–120_ *p*=0.338), ISR AUC_0–60_ (*p*=0.543) and ISR AUC_0–120_ (*p*=0.432) were similar between groups (Fig. 2c–f). There were no group differences in the glucose-ISR dose–response function (Fig. 2g), nor in model-derived components of beta cell function (Fig. 2h–j), including β-GS (*p*=0.280), β-RS (*p*=0.159), potentiation factor ratio (*p*=0.425) and ISR@5 (*p*=0.742; data not shown).

Insulin clearance was lower at fasting (−0.8±0.2 l min^−1^ m^−2^, *p*<0.0001) and during the OGTT (−0.3±0.1 l min^−1^ m^−2^, *p*=0.005) in individuals with MASLD than in the control group (Fig. 2k, l).

#### Glucoregulatory hormones and lipid substrates

**Fig. 3:**
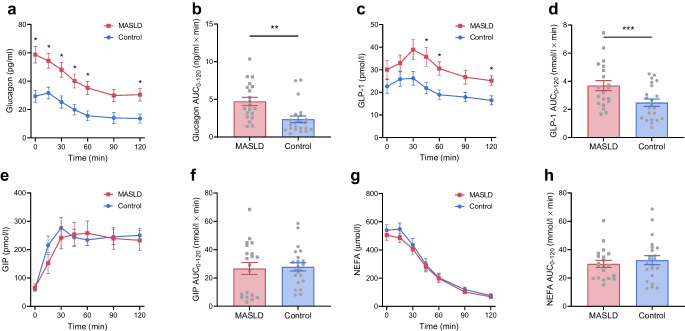
Glucoregulatory hormones and lipid substrates. Profiles and AUCs during a 75 g OGTT of plasma glucagon (**a**, **b**), GLP-1 (**c**, **d**), GIP (**e**, **f**) and NEFA (**g**, **h**) in individuals with early-stage MASLD (*n*=20) and control individuals (*n*=22). Data are mean ± SEM indicated by error bars. Group differences were analysed using Student’s *t* test. Repeated measures were analysed by two-way ANOVA followed by multiple pairwise comparisons using Sidak’s post hoc tests. *p<0.05, **p<0.01, ***p<0.001

Plasma glucagon levels were higher in the MASLD group than the control group during fasting (+29±7 pg/ml, *p*=0.0003) and throughout the OGTT (AUC_0–120_ +2.4±0.7 ng/ml × min, *p*=0.002) (Fig. 3a, b). Similarly, GLP-1 responses were consistently higher during the OGTT in the MASLD group (AUC_0–120_ +1.2±0.4 nmol/l × min, *p*=0.001) (Fig. 3c, d). Plasma GIP and NEFA concentrations were almost identical between groups during fasting and showed similar changes in response to the oral glucose load (AUC_0–120_ *p*=0.819 and *p*=0.521, respectively) (Fig. 3e–h).

#### Whole-body and tissue-specific insulin sensitivity

**Fig. 4:**
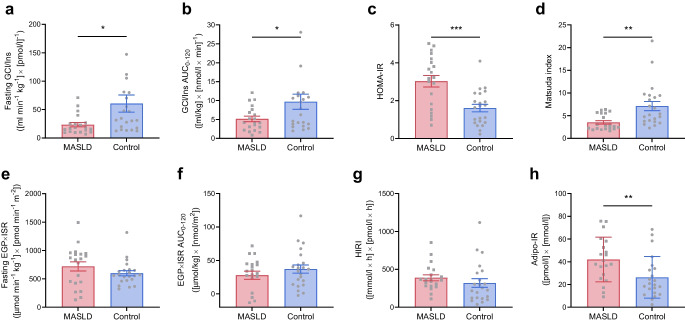
Whole-body and tissue-specific insulin sensitivity. (**a**–**d**) Whole-body insulin sensitivity measured by either tracer-derived glucose clearance normalised to plasma insulin levels (GCl/Ins) at fasting (**a**) and during a 75 g OGTT (**b**), or by HOMA-IR (**c**) and the Matsuda index (**d**) in individuals with early-stage MASLD (*n*=20) and control individuals (*n*=22). (**e**–**g**) Hepatic insulin resistance measured by either tracer-derived EGP × ISR, both at fasting (**e**) and during the OGTT (**f**), or by the HIRI (**g**). (**h**) Adipose tissue insulin resistance measured by the Adipo-IR index. Data are mean ± SEM indicated by error bars. Group differences were analysed using Student’s *t* test. *p<0.05, **p<0.01, ***p<0.001

In the fasted state and during the OGTT, participants with MASLD displayed lower whole-body insulin sensitivity than control participants as measured by either stable glucose isotopes (fasting GCl/Ins −37±16 [ml min^−1^ kg^−1^] × [nmol min^−1^ l]^−1^, *p*=0.025; GCl/Ins AUC_0–120_ −4.5±2.1 [ml/kg] × [nmol/l]^−1^, *p*=0.042, respectively), HOMA-IR (+1.4±0.4, *p*=0.001) or the Matsuda index (−3.6±1.2, *p*=0.004) (Fig. 4a–d).

There were no group differences in hepatic insulin resistance assessed by either glucose tracers (fasting EGP × ISR +119±92 μmol min^−1^ kg^−1^ × pmol min^−1^ m^−2^, *p*=0.207; EGP × ISR AUC_0–120_ μmol/kg × nmol/m^2^ −9.2±8.7, *p*=0.296) or the surrogate index (HIRI +70±69 [mmol/l×h] × [pmol/l × h], *p*=0.066) (Fig. 4e–g). Adipose tissue insulin resistance was higher in the MASLD group than in the control group (Adipo-IR +15.7±5.9, *p*=0.008) (Fig. 4h).

#### Associations between metabolic variables and prevalent MASLD

In univariable logistic regression analysis, oral glucose absorption was identified as a significant predictor of MASLD (Table 2). For each 1-SD increase in RaO AUC_0–60_, the OR for having MASLD was 2.11 (95% CI 1.04, 4.30, *p*=0.039). The receiver operating characteristic (ROC) analysis for RaO AUC_0–60_ in predicting MASLD yielded an AUC of 0.659. No significant associations were observed with whole-body insulin sensitivity (GCl/Ins AUC_0–120_) or hepatic insulin resistance (EGP × ISR AUC_0–120_).

**Table 2:** Multivariable associations between metabolic variables and prevalent MASLD

Variable	Univariable analysis	Multivariable model 1^a^	Multivariable model 2^b^	Multivariable model 3^c^
RaO AUC_0–60_	2.11 (1.04, 4.30)*	2.61 (1.10, 7.79)*	4.73 (1.53, 24.26)*	4.99 (1.44, 31.57)*
GCl/Ins AUC_0–120_	0.34 (0.10, 1.11)	0.17 (0.01, 1.04)	0.53 (0.04, 2.16)	1.32 (0.07, 18.33)
EGP × ISR AUC_0–120_	0.71 (0.37, 1.35)	0.43 (0.12, 1.21)	0.20 (0.03, 0.73)*	0.23 (0.02, 1.13)
Age	1.42 (0.75, 2.66)	1.01 (0.33, 3.07)	0.83 (0.21, 2.78)	1.30 (0.23, 8.07)
Sex, male	1.55 (0.45, 5.37)	4.61 (0.82, 36.23)	10.99 (0.81, 148.59)	12.52 (0.61, 257.97)
HbA_1c_	2.07 (1.03, 4.14)	1.10 (0.32, 4.17)	0.60 (0.14, 2.31)	0.33 (0.05, 1.53)
BMI	2.61 (1.22, 5.57)*	–	9.26 (1.89, 87.16)*	12.22 (1.92, 190.30)*
β-GS	0.70 (0.37, 1.34)	–	–	0.68 (0.19, 2.07)
Insulin clearance	0.27 (0.09, 0.79)*	–	–	0.22 (0.02, 1.42)

Data are presented as OR (95% CI) for MASLD for each 1-SD increase in the independent variable

^a^Model 1: adjusted for age, sex and HbA_1c_

^b^Model 2: as for model 1, with further adjustment for BMI

^c^Model 3: as for model 2, with additional inclusion of β-GS and insulin clearance as covariates

^*^p<0.05

The relationship between glucose absorption and MASLD remained statistically significant in multivariable models adjusted for age, sex and HbA_1c_ (OR 2.61 [1.10, 7.79], *p*=0.048), after further adjustment for BMI (OR 4.73 [1.53, 24.26], *p*=0.022), and after additional inclusion of β-GS and insulin clearance as covariates (OR 4.99 [1.44, 31.57], *p*=0.036) (Table 2). Furthermore, the association between RaO and prevalent MASLD was confirmed in BMI-matched subset analyses (ESM Table 2).

### Study 2

#### Participants

Ninety-one participants completed the OGTT with concurrent measurement of gastric emptying and were included in the analysis (48 men and 43 women, age 30 [23–40] years, BMI 24.6±3.4 kg/m^2^, HbA_1c_ 34.9±3.1 mmol/mol (5.3±0.3%), T50 85.6±28.8 min and HSI 33 [29–36]) (ESM Table 3). Compared with Study 1 participants, this cohort were of younger age and had different racial/ethnic composition, better glucose tolerance and lower BMI and HSI. Twenty-seven Study 2 participants had HSI ≥36, 38 participants had HSI between 30 and 36, and 26 participants had HSI <30. Their clinical and metabolic characteristics are reported in Table 3. Compared with participants with HSI <30, those with HSI ≥36 were older and had higher BMI, ALT and γ-glutamyl transferase levels. Sex distribution, fasting blood glucose, HbA_1c_, AST and alkaline phosphatase were comparable between groups.

**Table 3:** Clinical and metabolic characteristics of Study 2 participants

Characteristic	HSI <30 (*n*=26)	30≤ HSI <36 (*n*=38)	HSI ≥36 (*n*=27)	*p* value^a^
Women, *n* (%)	10 (38.5)	22 (57.9)	11 (40.7)	0.951
Age, years	27 (20–36)	30 (24–42)	36 (27–50)	0.022*
BMI, kg/m^2^	20.8±1.8	24.7±2.2	28.0±2.0	0.0001***
HbA_1c_, mmol/mol	34.5±2.5	34.8±3.0	35.2±3.8	0.413
HbA_1c_, %	5.3±0.2	5.3±0.3	5.4±0.4	0.418
Fasting blood glucose, mmol/l	5.1±0.5	5.1±0.4	5.3±0.5	0.296
1 h blood glucose, mmol/l	8.7±1.5	8.5±2.2	10.0±2.2	0.023*
2 h blood glucose, mmol/l	6.2±1.3	6.7±1.0	7.3±1.5	0.013*
AST, U/l	24 (21–30)	22 (20–25)	26 (22–30)	0.545
ALT, U/l	18 (13–22)	20 (16–27)	29 (23–47)	0.0001***
Alkaline phosphatase, U/l	75 (56–82)	68 (59–78)	78 (62–92)	0.400
γ-Glutamyl transferase, U/l	14 (11–19)	15 (11–19)	23 (15–36)	0.0001***
HSI	26.9±2.0	32.9±1.5	38.9±2.6	0.0001***
T50, min	86.5±32.3	89.8±23.5	78.7±31.6	0.378
Gastric retention 1 h post-OGTT, %	58.7±13.2	60.9±7.7	56.2±11.2	0.351

Data are presented as *n* (%), mean ± SD or median (IQR)

^a^Difference between subgroups with HSI≥36 and HSI<30

^*^p<0.05, ***p<0.001 (analysed by Fisher’s exact test, Student’s *t* test or Mann–Whitney test, as appropriate)

#### Glucose tolerance and gastric emptying

**Fig. 5:**
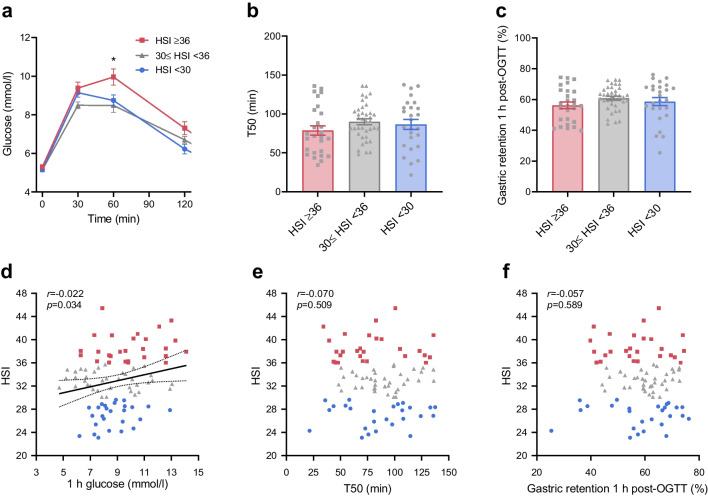
Relationships between glucose tolerance, gastric emptying and estimated hepatic steatosis. (**a**–**c**) Comparison of blood glucose responses to a 75 g OGTT (**a**), gastric half-emptying time (T50) (**b**) and gastric emptying at 1 h post-OGTT (**c**) between groups with HSI ≥36 (*n*=27), 30≤ HSI <36 (*n*=38) and HSI <30 (*n*=26). (**d**–**f**) Correlations of HSI with 1 h blood glucose (**d**), T50 (**e**) and gastric emptying at 1 h post-OGTT (**f**). Data are mean ± SEM. Group differences in blood glucose were analysed by two-way ANOVA. The differences in T50 and gastric emptying at 1 h post-OGTT between subgroups with HSI≥36 and HSI<30 were analysed using Student’s *t* test, with adjustment for age, sex, BMI and HbA_1c_. The relationships were evaluated using Pearson correlation. *p<0.05

After the glucose drink, the blood glucose response was higher in the group with HSI ≥36 (group effect: *p*=0.010) (Fig. 5a); this group showed higher blood glucose levels at 1 h and 2 h post-OGTT than the group with HSI <30 (Table 3).

Between these two groups, there were no differences in T50 or gastric retention (expressed as % of baseline) at 1 h post-OGTT, either before (*p*=0.378 and *p*=0.351, respectively) or after (*p*=0.760 and *p*=0.733) adjustment for age, sex, HbA_1c,_ and BMI (Fig. 5b, c).

The HSI was related directly to 1 h blood glucose (*r*=0.220, *p*=0.034) but was unrelated to the T50 (*p*=0.509) or to the gastric retention at 1 h post-OGTT (*p*=0.589) (Fig. 5d–f).

## Discussion

This study is the first to map postprandial glucose fluxes in individuals with early-stage MASLD. We demonstrated that, compared with healthy control individuals, individuals with MASLD and mild metabolic alterations, but without fibrosis, exhibited over 50% greater oral glucose absorption, resulting in higher plasma glucose and insulin responses to an oral glucose load. Moreover, early-stage MASLD was associated with impaired peripheral insulin sensitivity, marked by reduced GCl normalised to insulin and higher Adipo-IR, while hepatic insulin sensitivity and beta cell function remained largely preserved. Although gastric emptying is a key determinant of oral glucose absorption, we observed that there was no association between gastric emptying and estimated liver fat content in a second cohort of young, non-diabetic participants. Collectively, these observations support the concept that enhanced oral glucose absorption, likely at the intestinal level rather than due to rapid gastric emptying, represents an early feature of MASLD. Thus, glucose absorption appears to be a biologically plausible and actionable target for the prevention and management of MASLD.

Alterations in oral glucose handling marked by enhanced RaO may contribute to the onset and progression of MASLD through several interconnected mechanisms, as supported by preliminary clinical evidence [17, 18] and experimental studies [34]. Glucose absorbed from the gut is preferentially delivered to the liver via the portal vein, where excess glucose influx can stimulate triglyceride accumulation and promote MASLD progression from simple steatosis to steatohepatitis and fibrosis. Additionally, increased oral glucose absorption translates into higher plasma glucose excursions and sustained hyperinsulinaemia, which may further stimulate DNL and impair lipid export from the liver [34]. The molecular mechanisms may involve upregulation of key transcription factors such as carbohydrate-responsive element-binding protein (ChREBP) and sterol regulatory element-binding protein-1c (SREBP-1c), which upregulate the expression of lipogenic enzymes such as acetyl-CoA carboxylase (ACC) and fatty acid synthase (FAS) [35]. Rapid postprandial glucose elevation can also promote the formation of AGEs and reactive oxygen species (ROS), and hence stimulate the release of inflammatory cytokines [9, 34, 36]. Moreover, the persistent activation of DNL may generate lipotoxic intermediates such as diacylglycerols and ceramides, further activating inflammatory signalling pathways [34]. Oral glucose absorption can be modulated by a variety of nutritional [37–39], pharmacological [11, 15, 40] and surgical interventions [41], aimed at interfering with gastric emptying, carbohydrate breakdown and transporter expression or activity. A more comprehensive understanding of MASLD pathogenesis in relation to alterations in RaO will be crucial for informing the design of future targeted clinical trials.

The RaO is regulated by three interrelated mechanisms, namely gastric emptying, intestinal glucose absorption and splanchnic glucose uptake, which operate sequentially to modulate postprandial glucose flux. The finding that T50, as well as gastric retention at 1 h post-OGTT, was similar in individuals with low and high HSI suggests that gastric emptying is not a primary driver of liver fat accumulation. This narrows the focus towards alternative plausible mechanisms of elevated RaO in MASLD, particularly enhanced intestinal glucose absorption. Individuals with MASLD often consume high-carbohydrate, obesogenic diets, which may upregulate duodenal GLUT expression [42] and drive mucosal hyperplasia [43]. Supporting this view, prior clinical evidence shows that increased duodenal SGLT1 expression correlates with hepatic steatosis and fibrosis [9], whereas genetically reduced SGLT1 activity is linked to lower risk of MASLD [10]. Moreover, pharmacological SGLT1 inhibition prevents diet-induced steatosis in mice [11]. Splanchnic glucose uptake, representing first-pass glucose extraction by the gut and liver, has not been explored due to the difficulty of in vivo measurement [44]. Although reduced hepatic glucose uptake during hyperglycaemic clamp studies has been negatively associated with hepatic fat content [45], the specific contribution of splanchnic glucose uptake to oral glucose absorption in individuals with MASLD remains to be determined.

Early defects in insulin sensitivity are central to the pathogenesis of MASLD [3, 34]. However, emerging evidence challenges the notion that hepatic insulin resistance is the primary upstream event responsible for initiating intrahepatic fat accumulation. We observed that whole-body insulin actions on tracer-derived GCl were substantially impaired in early-stage MASLD, while hepatic responses to fasting and post-load insulin levels on EGP remained relatively intact, indicating peripheral rather than hepatic insulin resistance. Consistently, Sabatini et al [46] reported that tracer-measured EGP increases with biopsy-proven liver fibrosis and inflammation, driven by hepatic and extrahepatic insulin resistance, but not with the degree of steatosis. Likewise, Basset-Sagarminaga et al [45] did not find a significant association between insulin-induced EGP suppression and hepatic fat content. Differences in post-load EGP suppression in our study can be explained by higher plasma insulin levels in the MASLD group, resulting from reduced insulin clearance despite similarly stimulated insulin secretion, in line with previous evidence [4, 47]. This occurred alongside comparable declines in glucagon levels, which nevertheless remained consistently higher in the MASLD group. Hyperglucagonaemia has been consistently associated with hepatic steatosis and may reflect a disruption in the liver–alpha cell axis mediated by amino acids (i.e. glucagon resistance) [48]. On the contrary, identical NEFA levels throughout the OGTT exclude a substantial role for lipolytic control on gluconeogenesis in this setting, which is noteworthy given that EGP is largely influenced by NEFA flux to the liver [49].

An unexpected observation was the selective increase in total GLP-1 responses in MASLD, whereas GIP responses were comparable between groups. This dissociation argues against generalised incretin hypersecretion and instead suggests altered nutrient sensing or L cell-specific regulation. Given that GLP-1 and GLP-2 are co-secreted in equimolar amounts from enteroendocrine L-cells, enhanced GLP-1 secretion likely indicates concomitant augmentation of GLP-2 release. GLP-2 exerts well-established intestinotrophic effects and has been shown to increase mRNA expression of *SGLT1* (also known as *SLC5A1*) and glucose transport capacity, potentially via enhanced epithelial growth and enterocyte maturation along the crypt–villus axis [50]. Whether the amplified GLP-1/2 axis represents a compensatory adaptation to increased intestinal glucose absorption or contributes causally to altered intestinal–hepatic crosstalk in MASLD remains to be determined.

This study has some limitations that should be acknowledged. First, the cross-sectional design precludes establishing causality and temporal direction of the relationships between enhanced RaO and MASLD. Nonetheless, prior mechanistic evidence and current results provide a valuable framework for future longitudinal and interventional studies aimed at confirming and expanding these associations. Second, liver steatosis and fibrosis were assessed through methods that are either operator-dependent or indirect, and therefore subject to potential misclassification. To minimise variability and measurement bias, all ultrasound examinations were performed by experienced sonographers and surrogate indices were selected from those with well-established validation and strong support in clinical guidelines. Furthermore, hepatic steatosis was assessed using ultrasound in Study 1 and estimated using the HSI in Study 2. The use of different methods introduces heterogeneity that should be considered when interpreting cross-cohort observations. Third, the sample size was relatively small. However, it was adequate to detect clear-cut differences between groups, and the observed effect size supports the robustness of the main findings. Fourth, the study includes two independent cohorts with differing characteristics, especially with respect to BMI, preventing cross-cohort observations and the ability to draw conclusions about the relationship between RaO and gastric emptying in MASLD. Fifth, although associations persisted after BMI adjustment and matching, residual confounding by adiposity cannot be fully excluded. Finally, RaO AUC_0–120_ did not account for the entire 75 g oral glucose load, the remaining fraction likely reflecting ongoing absorption beyond 120 min, splanchnic extraction, temporary glycogen storage or metabolic conversion prior to systemic appearance, which have not been quantified. Thus, we cannot exclude that glucose uptake beyond the observation window ultimately contributes directly or indirectly to hepatic substrate availability.

In conclusion, our studies provide the first direct clinical evidence of enhanced RaO in people with early-stage MASLD, using a quasi-physiological dual-tracer method. The lack of apparent correlation between the gastric half-emptying time and estimated hepatic steatosis suggests that this phenomenon is likely to occur at the intestinal level, in concordance with prior histological evidence. These results uncover a novel pathogenetic mechanism and suggest a potential actionable target for early prevention and treatment of MASLD.

## Supplementary Information

Below is the link to the electronic supplementary material.

ESM (PDF 211 KB)

## Data Availability

The data that support the study findings are available from the corresponding authors on reasonable request starting from the date of publication and will remain accessible for a minimum of 10 years, in line with institutional policies. Access will be granted to qualified researchers to address research questions that are consistent with the scope of the original study and meet ethical and legal requirements.

## Abbreviations

β-GS: Beta cell glucose sensitivity
β-RS: Beta cell rate sensitivity
ALT: Alanine aminotransferase
AST: Aspartate aminotransferase
DNL: De novo lipogenesis
EGP: Endogenous glucose production
FIB-4: Fibrosis-4 index
GCl: Glucose clearance
GCl/Ins: Fasting GCl/plasma insulin ratio
GIP: Glucose-dependent insulinotropic polypeptide
GLP-1: Glucagon-like peptide-1
HIRI: Hepatic insulin resistance index
HSI: Hepatic steatosis index
ISR: Insulin secretion rate
ISR@5: ISR at a fixed glucose concentration of 5 mmol/l
MASLD: Metabolic dysfunction-associated steatotic liver disease
RaO: Rate of appearance of oral ingested glucose
SGLT1: Sodium–glucose cotransporter 1
SLD: Steatotic liver disease
T50: Gastric half-emptying time

## References

[1] Younossi ZM, Golabi P, Paik JM, Henry A, Van Dongen C, Henry L (2023) The global epidemiology of nonalcoholic fatty liver disease (NAFLD) and nonalcoholic steatohepatitis (NASH): a systematic review. Hepatology 77(4):1335–1347. 10.1097/hep.0000000000000004 36626630 10.1097/HEP.0000000000000004 PMC10026948

[2] Rinella ME, Lazarus JV, Ratziu V et al (2023) A multisociety Delphi consensus statement on new fatty liver disease nomenclature. Hepatology 78(6):1966–1986. 10.1097/hep.0000000000000520 37363821 10.1097/HEP.0000000000000520 PMC10653297

[3] Stefan N, Yki-Järvinen H, Neuschwander-Tetri BA (2025) Metabolic dysfunction-associated steatotic liver disease: heterogeneous pathomechanisms and effectiveness of metabolism-based treatment. Lancet Diabetes Endocrinol 13(2):134–148. 10.1016/s2213-8587(24)00318-8 39681121 10.1016/S2213-8587(24)00318-8

[4] Trico D, Caprio S, Rosaria Umano G et al (2018) Metabolic features of nonalcoholic fatty liver (NAFL) in obese adolescents: findings from a multiethnic cohort. Hepatology 68(4):1376–1390. 10.1002/hep.30035 29665034 10.1002/hep.30035 PMC6173637

[5] Trico D, Galderisi A, Mari A, Santoro N, Caprio S (2019) One-hour post-load plasma glucose predicts progression to prediabetes in a multi-ethnic cohort of obese youths. Diabetes Obes Metab 21(5):1191–1198. 10.1111/dom.13640 30663201 10.1111/dom.13640 PMC6459710

[6] Phillips LK, Deane AM, Jones KL, Rayner CK, Horowitz M (2015) Gastric emptying and glycaemia in health and diabetes mellitus. Nat Rev Endocrinol 11(2):112–128. 10.1038/nrendo.2014.202 25421372 10.1038/nrendo.2014.202

[7] Wu T, Rayner CK, Jones KL, Xie C, Marathe C, Horowitz M (2020) Role of intestinal glucose absorption in glucose tolerance. Curr Opin Pharmacol 55:116–124. 10.1016/j.coph.2020.10.017 33227625 10.1016/j.coph.2020.10.017

[8] Wright EM, Loo DD, Hirayama BA (2011) Biology of human sodium glucose transporters. Physiol Rev 91(2):733–794. 10.1152/physrev.00055.2009 21527736 10.1152/physrev.00055.2009

[9] Fiorentino TV, De Vito F, Suraci E et al (2022) Augmented duodenal levels of sodium/glucose co-transporter 1 are associated with higher risk of nonalcoholic fatty liver disease and noninvasive index of liver fibrosis. Diabetes Res Clin Pract 185:109789. 10.1016/j.diabres.2022.109789 35192912 10.1016/j.diabres.2022.109789

[10] Dobbie LJ, Cuthbertson DJ, Hydes TJ, Alam U, Zhao SS (2023) Mendelian randomisation reveals sodium-glucose cotransporter-1 inhibition’s potential in reducing non-alcoholic fatty liver disease risk. Eur J Endocrinol 188(6):K33–K37. 10.1093/ejendo/lvad068 37343141 10.1093/ejendo/lvad068 PMC11188739

[11] Honda Y, Ozaki A, Iwaki M et al (2021) Protective effect of SGL5213, a potent intestinal sodium-glucose cotransporter 1 inhibitor, in nonalcoholic fatty liver disease in mice. J Pharmacol Sci 147(2):176–183. 10.1016/j.jphs.2021.07.002 34384565 10.1016/j.jphs.2021.07.002

[12] Solini A, Trico D, Del Prato S (2023) Incretins and cardiovascular disease: to the heart of type 2 diabetes? Diabetologia 66(10):1820–1831. 10.1007/s00125-023-05973-w 37542009 10.1007/s00125-023-05973-w PMC10473999

[13] Drucker DJ, Holst JJ (2023) The expanding incretin universe: from basic biology to clinical translation. Diabetologia 66(10):1765–1779. 10.1007/s00125-023-05906-7 36976349 10.1007/s00125-023-05906-7

[14] Marathe CS, Rayner CK, Jones KL, Horowitz M (2013) Relationships between gastric emptying, postprandial glycemia, and incretin hormones. Diabetes Care 36(5):1396–1405. 10.2337/dc12-1609 23613599 10.2337/dc12-1609 PMC3631884

[15] Rayner CK, Watson LE, Phillips LK et al (2020) Effects of sustained treatment with lixisenatide on gastric emptying and postprandial glucose metabolism in type 2 diabetes: a randomized controlled trial. Diabetes Care 43(8):1813–1821. 10.2337/dc20-0190 32471908 10.2337/dc20-0190

[16] Trico D, Mengozzi A, Frascerra S, Scozzaro MT, Mari A, Natali A (2019) Intestinal glucose absorption is a key determinant of 1-hour postload plasma glucose levels in nondiabetic subjects. J Clin Endocrinol Metab 104(6):2131–2139. 10.1210/jc.2018-02166 30445459 10.1210/jc.2018-02166

[17] Andreozzi F, Mancuso E, Mazza E et al (2024) One-hour post-load glucose levels are associated with hepatic steatosis assessed by transient elastography. Diabetes Obes Metab 26(2):682–689. 10.1111/dom.15358 37953652 10.1111/dom.15358

[18] Jagannathan R, Fiorentino TV, Marini MA, Sesti G, Bergman M (2022) One-hour post-load glucose is associated with severity of hepatic fibrosis risk. Diabetes Res Clin Pract 189:109977. 10.1016/j.diabres.2022.109977 35772586 10.1016/j.diabres.2022.109977

[19] Vallet-Pichard A, Mallet V, Nalpas B et al (2007) FIB-4: an inexpensive and accurate marker of fibrosis in HCV infection. comparison with liver biopsy and fibrotest. Hepatology 46(1):32–36. 10.1002/hep.21669 17567829 10.1002/hep.21669

[20] Tacke F, Horn P, Wai-Sun Wong V et al (2024) EASL-EASD-EASO Clinical Practice Guidelines on the management of metabolic dysfunction-associated steatotic liver disease (MASLD). J Hepatol 81(3):492–542. 10.1016/j.jhep.2024.04.031 38851997 10.1016/j.jhep.2024.04.031

[21] American Diabetes Association (2026) 2. Diagnosis and classification of diabetes: standards of care in diabetes-2026. Diabetes Care 49(Supplement_1):S27–S49. 10.2337/dc26-S002 41358893 10.2337/dc26-S002 PMC12690183

[22] Gastaldelli A, Casolaro A, Pettiti M et al (2007) Effect of pioglitazone on the metabolic and hormonal response to a mixed meal in type II diabetes. Clin Pharmacol Ther 81(2):205–212. 10.1038/sj.clpt.6100034 17259945 10.1038/sj.clpt.6100034

[23] Mari A, Stojanovska L, Proietto J, Thorburn AW (2003) A circulatory model for calculating non-steady-state glucose fluxes. Validation and comparison with compartmental models. Comput Methods Programs Biomed 71(3):269–281. 10.1016/s0169-2607(02)00097-4 12799059 10.1016/s0169-2607(02)00097-4

[24] Van Cauter E, Mestrez F, Sturis J, Polonsky KS (1992) Estimation of insulin secretion rates from C-peptide levels. Comparison of individual and standard kinetic parameters for C-peptide clearance. Diabetes 41(3):368–377. 10.2337/diab.41.3.368 1551497 10.2337/diab.41.3.368

[25] Mari A, Ferrannini E (2008) Beta-cell function assessment from modelling of oral tests: an effective approach. Diabetes Obes Metab 10(Suppl 4):77–87. 10.1111/j.1463-1326.2008.00946.x 18834435 10.1111/j.1463-1326.2008.00946.x

[26] Trico D, Mengozzi A, Baldi S et al (2022) Lipid-induced glucose intolerance is driven by impaired glucose kinetics and insulin metabolism in healthy individuals. Metabolism 134:155247. 10.1016/j.metabol.2022.155247 35760117 10.1016/j.metabol.2022.155247

[27] Bizzotto R, Trico D, Natali A et al (2021) New insights on the interactions between insulin clearance and the main glucose homeostasis mechanisms. Diabetes Care 44(9):2115–2123. 10.2337/dc21-0545 34362813 10.2337/dc21-0545

[28] Trico D, Natali A, Mari A, Ferrannini E, Santoro N, Caprio S (2018) Triglyceride-rich very low-density lipoproteins (VLDL) are independently associated with insulin secretion in a multiethnic cohort of adolescents. Diabetes Obes Metab 20(12):2905–2910. 10.1111/dom.13467 30003666 10.1111/dom.13467 PMC6231949

[29] Abdul-Ghani MA, Matsuda M, Balas B, DeFronzo RA (2007) Muscle and liver insulin resistance indexes derived from the oral glucose tolerance test. Diabetes Care 30(1):89–94. 10.2337/dc06-1519 17192339 10.2337/dc06-1519

[30] Gastaldelli A, Harrison SA, Belfort-Aguilar R et al (2009) Importance of changes in adipose tissue insulin resistance to histological response during thiazolidinedione treatment of patients with nonalcoholic steatohepatitis. Hepatology 50(4):1087–1093. 10.1002/hep.23116 10.1002/hep.23116 19670459

[31] Sanaka M, Nakada K, Nosaka C, Kuyama Y (2007) The Wagner-Nelson method makes the [13C]-breath test comparable to radioscintigraphy in measuring gastric emptying of a solid/liquid mixed meal in humans. Clin Exp Pharmacol Physiol 34(7):641–644. 10.1111/j.1440-1681.2007.04624.x 17581222 10.1111/j.1440-1681.2007.04624.x

[32] Xie C, Huang W, Wang X et al (2021) Gastric emptying in health and type 2 diabetes: an evaluation using a 75 g oral glucose drink. Diabetes Res Clin Pract 171:108610. 10.1016/j.diabres.2020.108610 33301790 10.1016/j.diabres.2020.108610

[33] Lee JH, Kim D, Kim HJ et al (2010) Hepatic steatosis index: a simple screening tool reflecting nonalcoholic fatty liver disease. Dig Liver Dis 42(7):503–508. 10.1016/j.dld.2009.08.002 19766548 10.1016/j.dld.2009.08.002

[34] Loomba R, Friedman SL, Shulman GI (2021) Mechanisms and disease consequences of nonalcoholic fatty liver disease. Cell 184(10):2537–2564. 10.1016/j.cell.2021.04.015 33989548 10.1016/j.cell.2021.04.015 PMC12168897

[35] Smith GI, Shankaran M, Yoshino M et al (2020) Insulin resistance drives hepatic de novo lipogenesis in nonalcoholic fatty liver disease. J Clin Investig 130(3):1453–1460. 10.1172/jci134165 31805015 10.1172/JCI134165 PMC7269561

[36] Esposito K, Nappo F, Marfella R et al (2002) Inflammatory cytokine concentrations are acutely increased by hyperglycemia in humans. Circulation 106(16):2067–2072. 10.1161/01.Cir.0000034509.14906.Ae 12379575 10.1161/01.cir.0000034509.14906.ae

[37] Trico D, Baldi S, Tulipani A et al (2015) Mechanisms through which a small protein and lipid preload improves glucose tolerance. Diabetologia 58(11):2503–2512. 10.1007/s00125-015-3710-9 26224101 10.1007/s00125-015-3710-9

[38] Nesti L, Mengozzi A, Trico D (2019) Impact of nutrient type and sequence on glucose tolerance: physiological insights and therapeutic implications. Front Endocrinol (Lausanne) 10:144. 10.3389/fendo.2019.00144 30906282 10.3389/fendo.2019.00144 PMC6418004

[39] Yki-Järvinen H, Luukkonen PK, Hodson L, Moore JB (2021) Dietary carbohydrates and fats in nonalcoholic fatty liver disease. Nat Rev Gastroenterol Hepatol 18(11):770–786. 10.1038/s41575-021-00472-y 34257427 10.1038/s41575-021-00472-y

[40] Quast DR, Xie C, Bound MJ et al (2025) Effects of metformin on postprandial blood pressure, heart rate, gastric emptying, GLP-1, and prevalence of postprandial hypotension in type 2 diabetes: a double-blind placebo-controlled crossover study. Diabetologia 74(4):611–618. 10.2337/db24-0830 10.2337/db24-0830 39761379

[41] Liu H, Lefere S, Guillot A, Zheng M-H, Tacke F (2025) Bariatric surgery for metabolic dysfunction-associated steatotic liver disease (MASLD): current knowledge of mechanisms. Hepatology. 10.1097/hep.0000000000001417 40445858 10.1097/HEP.0000000000001417

[42] Lehmann A, Hornby PJ (2016) Intestinal SGLT1 in metabolic health and disease. Am J Physiol Gastrointest Liver Physiol 310(11):G887–G898. 10.1152/ajpgi.00068.2016 27012770 10.1152/ajpgi.00068.2016

[43] Aliluev A, Tritschler S, Sterr M et al (2021) Diet-induced alteration of intestinal stem cell function underlies obesity and prediabetes in mice. Nat Metab 3(9):1202–1216. 10.1038/s42255-021-00458-9 34552271 10.1038/s42255-021-00458-9 PMC8458097

[44] Basset-Sagarminaga J, van de Weijer T, Iozzo P, Schrauwen P, Schrauwen-Hinderling V (2023) Advances and challenges in measuring hepatic glucose uptake with FDG PET: implications for diabetes research. Diabetologia 67(3):407–419. 10.1007/s00125-023-06055-7 38099962 10.1007/s00125-023-06055-7

[45] Basset-Sagarminaga J, van de Weijer T, Koene E et al (2025) Hepatic steatosis is associated with impaired hepatic glucose uptake under hyperglycemic conditions, a dynamic whole-body (18)F-FDG PET approach. Diabetes Res Clin Pract 225:112288. 10.1016/j.diabres.2025.112288 40447140 10.1016/j.diabres.2025.112288

[46] Sabatini S, Sen P, Carli F et al (2024) Hepatic glucose production rises with the histological severity of metabolic dysfunction-associated steatohepatitis. Cell Rep Med 5(11):101820. 10.1016/j.xcrm.2024.101820 39566466 10.1016/j.xcrm.2024.101820 PMC11604487

[47] Trico D, Galderisi A, Mari A et al (2020) Intrahepatic fat, irrespective of ethnicity, is associated with reduced endogenous insulin clearance and hepatic insulin resistance in obese youths: a cross-sectional and longitudinal study from the Yale Pediatric NAFLD cohort. Diabetes Obes Metab 22(9):1628–1638. 10.1111/dom.14076 32363679 10.1111/dom.14076 PMC8174801

[48] WewerAlbrechtsen NJ, Færch K, Jensen TM et al (2018) Evidence of a liver–alpha cell axis in humans: hepatic insulin resistance attenuates relationship between fasting plasma glucagon and glucagonotropic amino acids. Diabetologia 61(3):671–680. 10.1007/s00125-017-4535-5 29305624 10.1007/s00125-017-4535-5

[49] Petersen MC, Vatner DF, Shulman GI (2017) Regulation of hepatic glucose metabolism in health and disease. Nat Rev Endocrinol 13(10):572–587. 10.1038/nrendo.2017.80 28731034 10.1038/nrendo.2017.80 PMC5777172

[50] Brubaker PL (2018) Glucagon-like peptide-2 and the regulation of intestinal growth and function. Compr Physiol 8(3):1185–1210. 10.1002/j.2040-4603.2018.tb00039.x 29978894 10.1002/cphy.c170055

