# Supplementary material for: Oral glucose absorption is enhanced in early metabolic dysfunction-associated steatotic liver disease
Source: Diabetologia. 2026 Apr 21;69(7):2073–87. doi: 10.1007/s00125-026-06730-5 (PMC13236804; doi:10.1007/s00125-026-06730-5)

Electronic supplementary material

Oral glucose absorption is enhanced in early metabolic dysfunction-associated steatotic liver disease

Domenico Tricò[1,2], Tongzhi Wu[3], Noemi Cimbalo[2,4], Martina Chiriacò[1,2,5], Cong Xie[3], Luca Sacchetta[2,4], Lorenzo Nesti[1,2], Simone Gallo[2,4], Lorenza Santoni[2,4], Maria Chiara Masoni[1,2], Giovanni Petralli[2,4], Teresa Vanessa Fiorentino[6], Roberto Bizzotto[7], Maria Tiziana Scozzaro[1,2], Silvia Frascerra[1,2], Simona Baldi[1,2], Maurizia Rossana Brunetto[1], Andrea Mari[7], Christopher K Rayner[3], Andrea Natali[1,2]

1. Department of Clinical and Experimental Medicine, University of Pisa, Pisa, Italy
2. Laboratory of Metabolism, Nutrition and Atherosclerosis, University of Pisa, Pisa, Italy
3. Adelaide Medical School and Centre of Research Excellence in Translating Nutritional Science to Good Health, The University of Adelaide, Adelaide, Australia
4. Department of Surgical, Medical and Molecular Pathology and Critical Care Medicine, University of Pisa, Pisa, Italy
5. Institute of Life Sciences, Sant'Anna School of Advanced Studies, Pisa, Italy
6. Department of Clinical and Molecular Medicine, University of Rome La Sapienza, Rome, Italy
7. Institute of Neuroscience, National Research Council, Padua, Italy

Correspondence:

- Domenico Tricò, Department of Clinical and Experimental Medicine, University of Pisa, Via Roma 67, Pisa, PI 56126, Italy; Tel: +39 050 993640; Fax: +39 050 553335; Email: domenico.trico@unipi.it
- Tongzhi Wu, Adelaide Health and Medical Sciences (AHMS) Building, Floor 6, North Terrace, Adelaide, SA 5000, Australia; Tel: +61 8 831 36535; Email: tongzhi.wu@adelaide.edu.au

ESM Table 1. Characteristics of Study 1 BMI-matched subset.

	**MASLD** (n=17)	**Control** (n=19)	***p* value**
Women, n (%)	6 (35)	8 (42)	0.742
Age, year	49 [32, 54]	47 [30, 56]	0.788
BMI, kg/m^2	27.6±3.6	25.9±3.7	0.177
HbA_{1c}, mmol/mol	37.6±5.4	35.6±4.2	0.213
HbA_{1c}, %	5.6±0.5	5.4±0.4	0.190
Fasting blood glucose, mmol/l	5.3±0.9	5.2±0.5	0.771
1h blood glucose, mmol/l	10.1±1.9	8.3±1.6	**0.005**
2h blood glucose, mmol/l	8.7±2.7	7.9±2.0	0.296
Aspartate aminotransferase (AST), IU/l	20 [18, 27]	17 [15, 24]	0.072
Alanine aminotransferase (ALT), IU/l	30 [17, 34]	16 [15, 24]	**0.037**
Alkaline phosphatase, IU/l	64 [47, 76]	78 [54, 91]	0.095
γ-Glutamyl transferase, IU/l	30 [16, 63]	14 [11, 24]	**0.023**
Hepatic steatosis index (HSI)	37 [33, 42]	33 [31, 38]	**0.026**

Data are number (%), mean±SD, or median [interquartile range]. Group differences were tested by Fisher's exact test, Student t test, or Mann-Whitney test, respectively. Statistically significant p values (<0.05) for are indicated in bold.

ESM Table 2. Multivariable associations between metabolic parameters and prevalent MASLD in Study 1 BMI-matched subset.

	**Univariable analysis**	**Multivariable model 1**	**Multivariable model 2**	**Multivariable model 3**
RaO $AUC_{0\text{-}60}$	**3.00 [1.38, 8.21]**	**6.52 [1.83, 43.87]**	**9.94 [2.21, 112.76]**	**11.59 [2.12, 164.72]**
GCl/Ins $AUC_{0\text{-}120}$	0.49 [0.13, 1.13]	0.15 [0.01, 1.12]	0.38 [0.02, 1.93]	0.85 [0.02, 14.65]
EGP×ISR $AUC_{0\text{-}120}$	0.67 [0.31, 1.31]	0.34 [0.07, 1.16]	0.16 [0.02, 0.75]	0.22 [0.01, 1.42]
Age	1.04 [0.52, 2.09]	0.65 [0.16, 2.27]	0.62 [0.15, 2.21]	0.83 [0.11, 6.11]
Sex [Male]	1.33 [0.35, 5.31]	**16.6 [1.57, 381.81]**	**32.86 [1.25, 866.44]**	38.52 [0.99, 1502.8]
HbA_{1c}	1.59 [0.80, 3.41]	0.95 [0.23, 4.26]	0.57 [0.12, 2.67]	0.31 [0.03, 1.82]
BMI	1.84 [0.78, 4.69]	–	5.86 [0.88, 64.79]	7.87 [0.96, 128.71]
Beta cell glucose sensitivity	0.73 [0.35, 1.40]	–	–	0.63 [0.14, 2.21]
Insulin clearance	**0.31 [0.09, 0.75]**	–	–	0.27 [0.02, 1.91]

Data are odds ratio [95% confidence interval] for MASLD for each 1-SD increase in the independent variable. Statistically significant p values (<0.05) are indicated in bold.

ESM Table 3. Characteristics of Study 1 and Study 2 participants.

	**Study 1** (n=42)	**Study 2** (n=91)	***p* value**
Women, n (%)	17 (41)	43 (47)	0.574
Age, year	47 [30, 56]	30 [23, 40]	**<0.0001**
Race/Ethinicity*			**<0.0001**
Non-Hispanic White	42 (100)	40 (44)	
Asian	0 (0)	44 (48)	
Others	0 (0)	7 (8)	
BMI, kg/m^2	26.7±4.6	24.6±3.4	**0.008**
HbA_{1c}, mmol/mol	36.7±4.9	34.9±3.1	**0.033**
HbA_{1c}, %	5.5±0.4	5.3±0.3	**0.027**
Fasting blood glucose, mmol/l	5.3±0.7	5.2±0.5	0.361
1h blood glucose, mmol/l	9.2±1.8	9.0±2.1	0.595
2h blood glucose, mmol/l	8.4±2.3	6.7±1.4	**<0.0001**
Aspartate aminotransferase (AST), IU/l	19 [15, 24]	24 [21, 28]	**0.0002**
Alanine aminotransferase (ALT), IU/l	17 [15, 30]	20 [16, 29]	0.335
Alkaline phosphatase, IU/l	67 [47, 81]	74 [58, 80]	0.119
γ-Glutamyl transferase, IU/l	20 [11, 37]	16 [13, 23]	0.277
Hepatic steatosis index (HSI)	35 [32, 41]	33 [29, 36]	**0.015**

Data are number (%), mean±SD, or median [interquartile range]. Group differences were tested by Fisher's exact test, Student t test, or Mann-Whitney test, respectively. Statistically significant p values (<0.05) for are indicated in bold.
* Self-reported.

ESM Figure 1. Plasma fractional enrichment of 6,6-[$^2H_2$]glucose infused intravenously during a 75g oral glucose tolerance test.

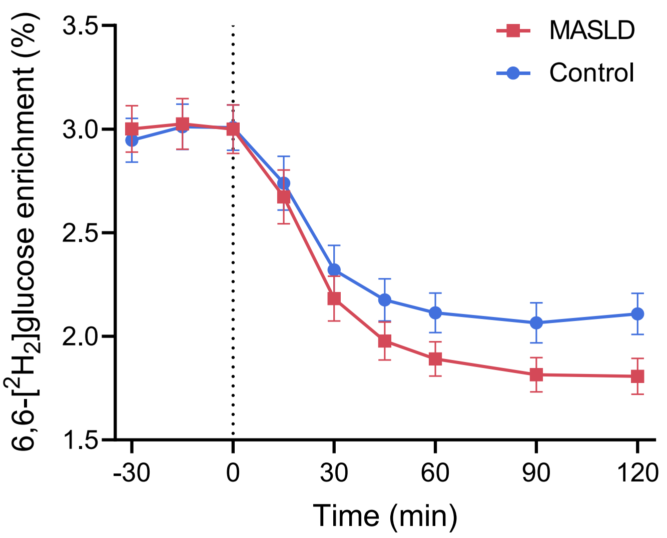

Supplement: Supplementary file 1 — ESM (PDF 211 KB) [file 125_2026_6730_MOESM1_ESM.pdf]
